# Correlation between egfr expression and accelerated proliferation during radiotherapy of head and neck squamous cell carcinoma

**DOI:** 10.1186/1748-717X-7-143

**Published:** 2012-08-24

**Authors:** Piernicola Pedicini, Antonio Nappi, Lidia Strigari, Barbara Alicia Jereczek-Fossa, Daniela Alterio, Marta Cremonesi, Francesca Botta, Barbara Vischioni, Rocchina Caivano, Alba Fiorentino, Giuseppina Improta, Giovanni Storto, Marcello Benassi, Roberto Orecchia, Marco Salvatore

**Affiliations:** 1 I.R.C.C.S. C.R.O.B.Regional Cancer Hospital, Rionero in Vulture, Italy; 2 Regina Elena National Cancer Institute, Roma, Italy; 3 I.E.O. European Institute of Oncology, Milan, Italy; 4 University of Milan, Milan, Italy; 5 Scientific Institute of Tumours of Romagna I.R.S.T, Meldola, Italy; 6 I.R.C.C.S SDN Foundation, Naples, Italy; 7 Division of Radiation Oncology and Radiobiology, National Center for Oncological Hadrontherapy CNAO, Pavia, Italy

**Keywords:** EGFr, Doubling time, Potential doubling time, Cell loss factor

## Abstract

**Purpose:**

To investigate the correlation between the expression of Epidermal Growth Factor receptor (EGFr) and the reduction of the effective doubling time (*T*_*D*_) during radiotherapy treatment and also to determine the dose per fraction to be taken into account when the overall treatment time (OTT) is reduced in accelerated radiotherapy of head and neck squamous cell carcinoma (HNSCC).

**Methods:**

A survey of the published papers comparing 3-years of local regional control rate (LCR) for a total of 2162 patients treated with conventional and accelerated radiotherapy and with a pretreatment assessment of EGFr expression, was made. Different values of *T*_*D*_ were obtained by a model incorporating the overall time corrected biologically effective dose (*BED*) and a 3-year clinical LCR for high and low EGFr groups of patients (H_EGFr_ and L_EGFr_), respectively. By obtaining the *T*_*D*_ from the above analysis and the sub-sites’ potential doubling time (*T*_*pot*_) from flow cytometry and immunohistochemical methods, we were able to estimate the average *T*_*D*_ for each sub-site included in the analysis. Moreover, the dose that would be required to offset the modified proliferation occurring in one day (*D*_*prolif*_), was estimated.

**Results:**

The averages of *T*_*D*_ were 77 (27-90)_95%_ days in L_EGFr_ and 8.8 (7.3-11.0)_95%_ days in H_EGFr_, if an onset of accelerated proliferation *T*_*K*_ at day 21 was assumed. The correspondent H_EGFr_ sub-sites’ *T*_*D*_ were 5.9 (6.6), 5.9 (6.6), 4.6 (6.1), 14.3 (12.9) days, with respect to literature immunohistochemical (flow cytometry) data of *T*_*pot*_ for Oral-Cavity, Oro-pharynx, Hypo-pharynx, and Larynx respectively. The *D*_*prolif*_ for the H_EGFr_ groups were 0.33 (0.29), 0.33 (0.29), 0.42 (0.31), 0.14 (0.15) Gy/day if *α* = 0.3 Gy^-1^ and *α/β* = 10 Gy were assumed.

**Conclusions:**

A higher expression of the EGFr leads to enhanced proliferation. This study allowed to quantify the extent of the effect which EGFr expression has in terms of reduced *T*_*D*_ and *D*_*prolif*_ for each head and neck sub-site.

## Background

HNSCC accelerates the production of clonogenic cells during radiotherapy, whereby an amount of a given dose of radiation may be used to sterilize cells produced during the treatment [1]. Therefore, by maintaining the same total dose, a reduction of OTT results in increased T-site control.

The benefit of reduced OTT has been tested in several studies comparing conventional treatment with accelerated fractionation schedules. The data showed an improved 5-year LCR [2,3].

However, the response is heterogeneous with respect to the different expressions of EGFr in the patient population and also to the sub-sites, as accelerated repopulation of clonogenic tumour cells and locoregional control could arise.

EGFr is overexpressed in the majority of HNSCC [4] and activation of the receptor leads to phosphorylation of the tyrosine kinase domains on the intracellular part of the receptor, activating downstream cascades which result in altered gene activation and modulation of the cell products. This has been related to increased cell proliferation, decreased apoptotic activity, increased angiogenesis, increased invasive and metastatic potential, and hence increased resistance to anti tumour therapy.

Furthermore, it has been shown that tumours with high expression of EGFr have a better LCR when treated with accelerated radiotherapy, while there was no benefit of acceleration in tumours with low EGFr.

Consequently, high EGFr has been suggested as a negative prognostic factor when OTT is prolonged, and as a positive prognostic factor when treatment time is reduced [5].

The aim of the present study is to investigate the correlation between EGFr expression and the reduction of *T*_*D*_ during radiotherapy treatment and also to determine the dose per fraction to be taken into account when the OTT is reduced in accelerated radiotherapy of HNSCC.

To achieve this goal, the data published in the literature were reviewed and analyzed by comparing different 3-year LCR and OTT for various dose fractionation schemes, also taking into account different sub-sites of HNSCC.

## Methods

### Literature review

The primary end point considered for the present analysis was LCR, defined as the probability of avoiding local regional recurrence of cancer at the primary tumour site (T) or nodal (N) position, within 3-years after the end of radiotherapy.

A survey of the published papers comparing LCR for patients with HNSCC treated with conventional and accelerated radiotherapy, respectively, and with a pretreatment assessment of EGFr expression, was made [6-11].

In the published papers, different criteria of EGFr expression assessment according to the intensity of staining were used. EGFr expression was classified by the investigators, with several quantitative or semi-quantitative scoring systems, i.e., absent, minimal, moderate, or intense staining (Table 1). The main characteristics for selection were conventional and accelerated fractionations, different OTT, assessment of EGFr expression and LCR, as listed in Table 1.

**Table 1:** Treatment characteristics of the selected group

**Author**	**Fractionation**	**OTT(days)**	**SI**_**H**_**%**	**SI**_**L**_**%**	**LCR**_**H**_	**LCR**_**L**_	**HR**	**S**
Eriksen AO a [6]	33x2Gy	66	SI ≥ 50	SI < 50	0.15	0.44	Y	N
Eriksen AO b [6]	33x2Gy	45	SI ≥ 50	SI < 50	0.64	0.55	Y	N
Eriksen AO c [6]	33x2Gy	38	SI ≥ 50	SI < 50	0.77	0.57	Y	N
Eriksen RO a [7]	33x2Gy	45	SI ≥ 50	SI < 50	0.57	0.63	Y	N
Eriksen RO b [7]	33x2Gy	38	SI ≥ 50	SI < 50	0.70	0.62	Y	N
Bentzen JCO a [8]	33x2Gy	45	SI ≥ 40	SI < 40	0.30	0.45	N	N
Bentzen JCO b [8]	36x1.5 Gy	12	SI ≥ 40	SI < 40	0.54	0.49	N	N
Suwinski IJROBP a [9]	35x1.8 Gy	47	SI ≥ 33	SI < 33	0.33	0.70	N	Y
Suwinski IJROBP b [9]	35x1.8 Gy	35	SI ≥ 33	SI < 33	0.58	0.73	N	Y
Smid IJROBP a [10]	25x2Gy + 5x2.5 Gy	~ 46	A/m	M/I	0.69	0.65	N	Y
Smid IJROBP b [10]	25x2Gy + 5x2.5 Gy	~ 34	A/m	M/I	0.91	0.68	N	Y
Chung IJROBP a [11]	35x2Gy	47	SI ≥ 80	SI < 80	0.36	0.61	N	N
Chung IJROBP b [11]	30x1.8 Gy + 12x1.5 Gy	38	SI ≥ 80	SI < 80	0.54	0.68	N	N

*Abbreviations*: *SI* = Staining Intensity cut-point; *A/m* = Absent/minimal; *M/I* = Moderate/Intense; *HR* = Hypoxic Radiosensitizer; *S* = Surgery.

Only those studies which reported a median follow-up of at least 3-years were included in the analysis. Table 2 lists the main clinical characteristics of the patients, namely age, sex, primary site, T stage and N stage. Further clinical information are in the reviewed papers.

**Table 2:** Clinical characteristics of the selected groups

**Author**		**E1**	**E2**	**BE**	**SU**	**SM**	**CH**
**number of patients**		209	803	304	148	165	533
**Sex %**	M	74.2	81.0	72.0	90.0	66.0	79.2
	F	25.8	19.0	28.0	10.0	34.0	20.8
**Primary site %**	Oral Cavity	/	12.0	13.0	50.6	100.0	10.3
	Oropharynx	/	52.5	28.0		/	60.4
	Hypopharynx	/		12.0	/	/	13.7
	Nasopharynx	/	/	3.0	/	/	/
	Larynx	100.0	35.5	44.0	49.4	/	15.6
**T stage %**	T1	6.0	67.0	3.0	26.1	2.0	5.8
	T2	37.0		42.0		25.0	27.6
	T3	35.0	33.0	33.0	73.9	28.0	37.1
	T4	22.0		22.0		35.0	29.3
	Tx	/	/	/	/	/	0.2
**N stage %**	N0	65.0	65.0	63.0	33.8	26.0	21.8
	N1	35.0	35.0	18.0	66.2	16.0	18.6
	N2a			15.0		1.0	9.6
	N2b					39.0	17.8
	N2c					17.0	19.3
	N3			4.0		1.0	12.9

*Abbreviations*: *E1* = Eriksen-AO, *E2* = Eriksen RO, *BE* = Bentzen JCO, *SU* = Suwinski IJROBP, *SM* = Smid IJROBP, *CH* = Chung IJROBP.

### Radiobiological analysis

The tumour effects were evaluated by the overall time corrected *BED* as in eq. (1)

(1) $$BED=nd\left(1+\frac{d}{\alpha /\beta }\right)-\frac{\ln2}{\alpha }\frac{\left(T-T_{k}\right)}{T_{D}}$$

where *n* is the number of fractions of size *d* in Gy, *α* and *β* are the linear quadratic coefficients of dose, *T* is the overall time, *T*_*k*_ is the onset time for accelerated proliferation and *T*_*D*_ the effective doubling time. The first term in eq. (1) (the dosimetric component, see Appendix A), is affected by differences in EGFr expression because of modification to *α* and *β* parameters that describe the intrinsic and repair radiosensitivity of tumour types, respectively. We add the subscripts *H* and *L* to indicate high or low EGFr expression respectively (*BED*_*H*_*(d)* or *BED*_*L*_*(d)*). The second term (the temporal component, see Appendix A) is affected by differences in EGFr expression due to the presence of the *α* parameter (*α*_*H*_ or *α*_*L*_) and *T*_*D*_ (*T*_*DH*_ or *T*_*DL*_). Superscripts *S* and *F* are specified to distinguish between conventional (*S = Slow*) and accelerated (*F = Fast*) fractionations, respectively.

From *BED* we have the standard model of tumour control probability (*TCP*) using the linear-quadratic model incorporating the Poisson’s low [12],

(2) $$\begin{array}{l} TCP=\exp\left(-N\cdot S\right)\\ \;=\exp\left[-N\cdot \exp\left(-\alpha \cdot BED\right)\right] \end{array}$$

where *N* = *ρ·V* (*ρ* = cell density and *V* = volume) represents the initial number of potential proliferating cells in the tumour. Therefore, the cell survival probability being *S = exp(−α·BED)*, the *TCP* represents the probability of avoiding local recurrence [13] at total dose *D = n·d* whereby we write *TCP* = LCR.

Moreoever, in order to analyze the effects of EGFr expression due to the change in the OTT, the papers chosen in the survey had the same dose per fraction and total dose but a different OTT.

Thus, by taking the natural logarithms of eq. (2) written for fast and slow fractionations, dividing the resultant equations and by taking the natural logarithm again, we get

(3.a) $$\ln\left(\frac{\ln LCR_{H}^{S}}{\ln LCR_{H}^{F}}\right)=\frac{\ln2}{T_{\mathrm{DH}}}\left(T^{S}-T^{F}\right);$$

for high EGFr expression group, and

(3.b) $$\ln\left(\frac{\ln LCR_{L}^{S}}{\ln LCR_{L}^{F}}\right)=\frac{\ln2}{T_{\mathrm{DL}}}\left(T^{S}-T^{F}\right);$$

for low EGFr expression group (see Appendix A).

This expedient allows to eliminates the dependence of findings from the choice of dose fractionation and from the estimated values of *α* and *β*. The equations (3.a) and (3.b) are also independent notwithstanding the assumption about number of cells *N*. The uncertainties arising from these assumptions strongly influences the results of the other models that depend on such parameters. Therefore, this is the main advantage of equations (3.a) and (3.b).

**Figure 1:**
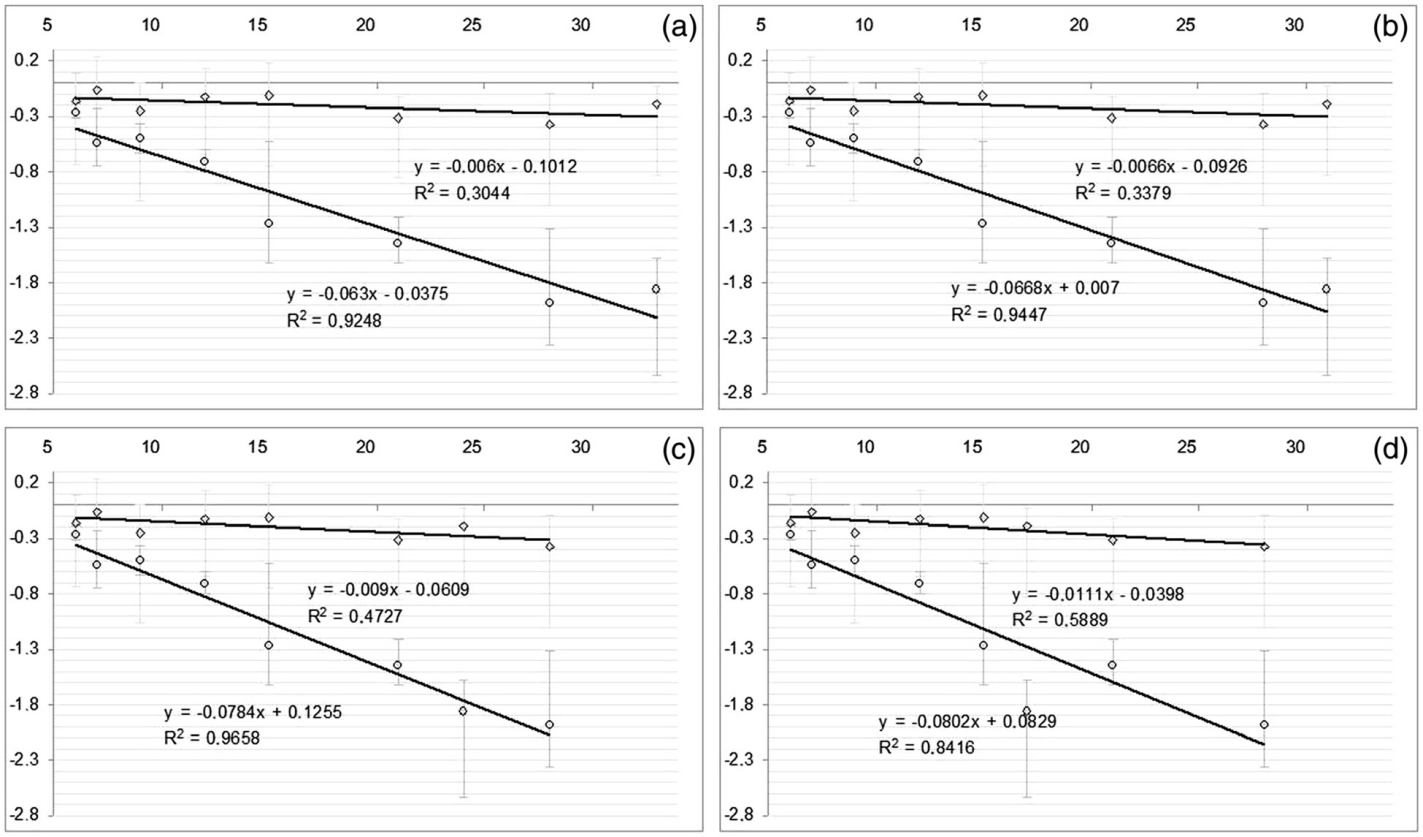
**Linear regressions for L**_**EGFr**_**group (diamonds) and H**_**EGFr**_**group (circles) with *y*** **=** ***ln(ln*LCR**^***S***^***/*lnLCR**^**F**^***) *and *x = (T***^***S***^***-T***^***F***^***) *(days).** The higher angular coefficient of H_EGFr_ group line demonstrate a reduction of effective doubling time with respect to L_EGFr_ group (proportionality with the inverse of the effective doubling time). Sub-figures refer to a different onset of accelerated repopulation (**a**) *T*_*k*_ = 0, (**b**) *T*_*k*_ = 14, (**c**) *T*_*k*_ = 21, (**d**) *T*_*k*_ = 28 days. Error bars represent the 95% confidence intervals.

In each of these equations appears only one unknown (the effective doubling time) for which, being in a linear form, they are suitable for an easy comparison between LCR due to different EGFr expression groups with different OTT. This assessment was done by evaluating the differences of angular coefficients (ln2/*T*_*DH*_*vs* ln2/*T*_*DL*_) from the correspondent regression lines obtained by LCR available in literature (Figure 1). For those papers, where in addition to differences of OTT there are also differences in terms of dose fractionation, the correction as described in Appendix B was done.

**Figure 2:**
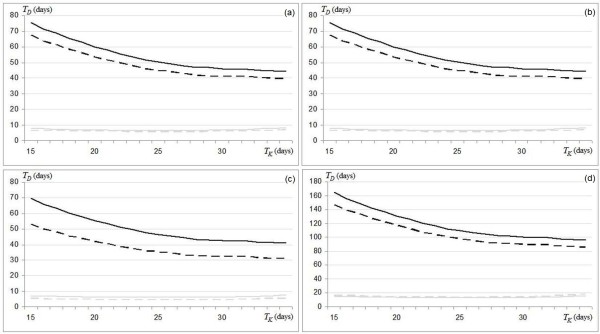
**Curves of *T***_***D***_**obtained by varying *T***_***k***_**, for H**_**EGFr**_**(black) and L**_**EGFr**_**(gray) groups based on the immunohistochemistry (continuous) and flow cytometry (dashed) methods to estimate the sub-sites **^***i***^***T***_***pot***_**.** Differences are shown for Oral-Cavity (**a**), Oropharynx (**b**), Hypopharynx (**c**) and Larynx (**d**).

Furthermore, dividing equations (3.a) and (3.b), we also obtained the ratio of the actual doubling times between the H_EGFr_ and L_EGFr_ groups that allows a direct analysis of the EGFr effects (Figure 2) as follows

(4) $$T_{\mathrm{DL}}/T_{\mathrm{DH}}=ln\left(\frac{lnLCR_{H}^{S}}{lnLCR_{H}^{F}}\right)/\ln\left(\frac{lnLCR_{L}^{S}}{lnLCR_{L}^{F}}\right)$$

### Clinical analysis

The actual doubling times obtained from the above analysis, represent a weighted average of the doubling times from different sub-sites as oral cavity (18.2% of patients), oro-pharynx (30.3% of patients), hypo-pharynx (14.8% of patients), and larynx (36.4% of patients).

These sub-sites contribute differently to the average *T*_*D*_ because they have different *T*_*pot*_. However, the *T*_*D*_ for each sub-sites can be estimated if *T*_*pot*_ and the cell loss factor (*ϕ*) are known as described by Steel [14]. In particular *T*_*pot*_ can be measured by a single biopsy with flow cytometry as well as immunohistochemistry techniques.

**Table 3:** Numerical results of sub-site

** Sub-site**	**Number of patients**	**Doubling time**	**Flow Cytometric (FCM)**				**Immunohistochemical (Hi)**			
			**days**	**D**_**p**__**(0.25)**_	**D**_**p**__**(0.3)**_	**D**_**p**__**(0.35)**_	**days**	**D**_**p**__**(0.25)**_	**D**_**p**__**(0.3)**_	**D**_**p (0.35)**_
Oral Cavity	394 (18.2%)	**T**_**D**_	6.6 (5.5-8.2)	0.35 (0.28-0.42)	0.29 (0.23-0.35)	0.25 (0.20-0.30)	5.9 (4.9-7.4)	0.39 (0.31-0.47)	0.33 (0.26-0.39)	0.28 (0.22-0.34)
		**T**_**pot**_	3.9	0.59	0.49	0.42	2.3	1.00	0.84	0.72
Oropharynx	655 (30.3%)	**T**_**D**_	6.6 (5.5-8.2)	0.35 (0.28-0.42)	0.29 (0.23-0.35)	0.25 (0.20-0.30)	5.9 (4.9-7.4)	0.39 (0.31-0.47)	0.33 (0.26-0.39)	0.28 (0.22-0.34)
		**T**_**pot**_	3.9	0.59	0.49	0.42	2.3	1.00	0.84	0.72
Hypopharynx	320 (14.8%)	**T**_**D**_	6.1 (5.1-7.6)	0.38 (0.30-0.45)	0.31 (0.25-0.38)	0.27 (0.22-0.32)	4.6 (3.9-5.8)	0.50 (0.40-0.59)	0.42 (0.33-0.49)	0.36 (0.28-0.42)
		**T**_**pot**_	3.6	0.64	0.53	0.46	1.8	1.28	1.07	0.92
Larynx	784 (36.3%)	**T**_**D**_	12.9 (10.7-16.0)	0.18 (0.14-0.22)	0.15 (0.12-0.18)	0.13 (0.10-0.15)	14.3 (12.4-18.1)	0.16 (0.13-0.19)	0.14 (0.11-0.16)	0.12 (0.9-0.13)
		**T**_**pot**_	7.6	0.30	0.25	0.46	5.6	0.41	0.34	0.29

*T*_*D*_ (days) and *D*_*prolif*_ (Gy/day) for high EGFr expression group (<^*i*^***T***_*pot*_ **>** =5.2 days by flow cytometry; <^*i*^***T***_*pot*_ **>** =3.4 days by immunohistochemistry; *T*_*K*_ = 21 days). The ^*i*^*T*_*D*_ for each *i* sub-site (upper rows) is almost twice of ^*i*^*T*_*pot*_ by flow cytometry and more than double of ^*i*^*T*_*pot*_ by immunohistochemistry from literature (bottom rows) [15]. Consequently, a preclinical estimation of *D*_*prolif*_ by flow cytometry (left columns, bottom rows) or immunohistochemistry (right columns, bottom rows), results almost double and more than double if compared to the estimation of *D*_*prolif*_ obtained by mathematical model (left and right columns, upper rows), respectively. The 95 % confidence intervals are shown within the brackets.

*Abbreviations*: ^*i*^*D*_*p*(0.25; 0.3; 0.35)_ = *D*_*prolif*_ calculated with *α* = 0.25, *α* = 0.3, *α* = 0.35 Gy^-1^ respectively, *α/β* = 10 Gy, *d* = 2 Gy.

Therefore, the average cell loss factor was estimated using pretreatment data about *T*_*pot*_ available in literature [15,16], then the actual doubling time for each *i* sub-site (^*i*^*T*_*D*_) was obtained (see Appendix C).

Moreover, from ^*i*^*T*_*D*_ we also estimated the dose (in fractions of size *d*) that would be required to offset the effect of proliferation occurring in one day [17] by the follows equation

(5) $${}^{i}D_{\text{prolif}}=\frac{\ln2}{{}^{i}T_{D}\left(\alpha +\beta d\right)}$$

### Statistical analysis

In all the original studies of the survey the primary endpoint was LCR, 3 or 5-years after completion of radiotherapy, although only the 3-year LCR were extrapolated in order to compare the homogeneous parameters. LCR were assessed by the Kaplan-Meier method with a log rank test (statistical significance: *p* ≤ 0.05, two-sided). The LCR 95% confidence intervals are obtained by Greenwood’ formula [18]. Comparison between regression lines was done by Fisher’s exact test.

## Results

Table 1 and 2 describe the main clinical characteristics and treatment parameters of the selected groups in the survey.

Linear regression lines from equations (3.a) and (3.b), for H_EGFr_ and L_EGFr_ groups, are shown in Figure 1 with respect to different choices of the onset for accelerated repopulation (*T*_*k*_). The significant distinction of the angular coefficients for different groups (*p-values ≤* 0.02) correspond to an average *T*_*D*_ of 77 days (27–90)_95%_ for L_EGFr_ and to an average of 8.8 days (7.3-11.0)_95%_ for H_EGFr_, if an onset of accelerated proliferation *T*_*K*_ at day 21 was assumed.

In Figure 2 the significant H_EGFr_*T*_*D*_ reduction with respect to L_EGFr_*T*_*D*_ for each head and neck sub-site, are shown by varying *T*_*K*_.

**Figure 3:**
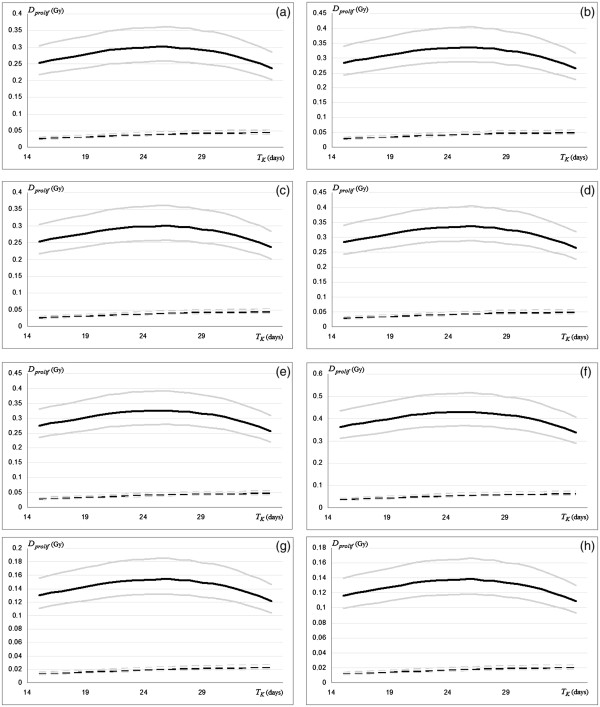
**Curves of *D***_***prolif***_**obtained by varying *T***_***k***_**, for H**_**EGFr**_**(continuous) and L**_**EGFr**_**(dashed) groups based on the flow cytometry (left column) and immunohistochemistry (right column) methods to estimate the sub-sites **^***i***^***T***_***pot***_**.** Sensitivity analysis is shown with respect to different values of *α* with *α/β* = 10 Gy: *α* = 0.25 Gy^-1^ (upper curves); *α* = 0.3 Gy^-1^ (mean curves); *α* = 0.35 Gy^-1^ (bottom curves). Differences are shown for Oral-Cavity (**a**,**b**), Oropharynx (**c**,**d**), Hypopharynx (**e**,**f**) and Larynx (**g**,**h**).

In Figure 3 the averages of *D*_*prolif*_ are shown based on the flow cytometry and immunohistochemical methods to estimate the sub-sites ^*i*^*T*_*pot*_. The maximum value of *D*_*prolif*_, up to about 0.5 Gy/day, is obtained corresponding to an onset of accelerated repopulation that starts from the fourth week (*T*_*K*_ at about 28^th^ day). Sensitivity analysis is shown with respect to different values of *α* with *α/β* = 10 Gy.

The weighted average potential doubling times < ^*i*^*T*_*pot*_ > of 5.2 days and 3.4 days [15,16] were obtained corresponding to averages for cell loss factors as < *ϕ*_(FCM)_ > = 0.41 (0.29-0.52)_95%_ and < *ϕ*_(Hi)_ > = 0.61 (0.53-0.69)_95%_ with respect to the flow cytometry and immunohistochemistry, respectively.

Table 3 reports numerical results for each sub-site in H_EGFr_ group with ^*i*^*T*_*D*_ and ^*i*^*D*_*prolif*_ calculated for different values of *α* (*α/β* = 10 Gy). It may be noted that the ^*i*^*T*_*D*_ for each *i* sub-site is almost twice of ^*i*^*T*_*pot*_ obtained by flow cytometry and more than double of ^*i*^*T*_*pot*_ obtained by immunohistochemistry. This means that a pre-treatment assessment of *D*_*prolif*_ by flow cytometry or immunohistochemistry may significantly overestimate the dose required to offset the accelerated proliferation occurring in one day.

**Figure 4:**
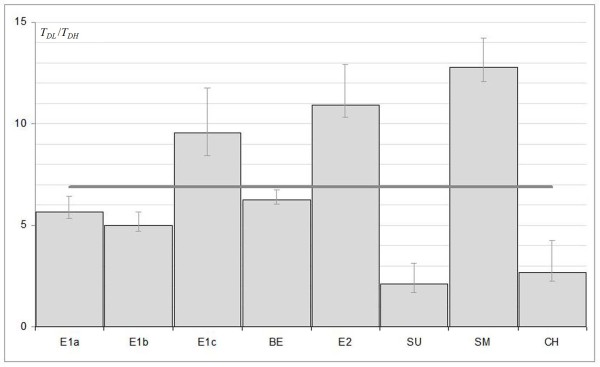
**Histogram of ratios between *T***_***D***_**values in the L**_**EGFr**_**and H**_**EGFr **_**groups.** The error bars represent the 95% confidence intervals while the overlay trend line represent the average of values. Abbreviations: E1 = Eriksen from AO, E2 = Eriksen RO, BE = Bentzen JCO, SU = Suwinski IJROBP, SM = Smid IJROBP, CH = Chung IJROBP.

In Figure 4, the histogram of the ratio between *T*_*DL*_ and *T*_*DH*_ (eq. 4) shows an average reduction of about 7 times in average (6.6-8.3)_95%_ for the H_EGFr_ group with respect to the L_EGFr_. This ratio could have significant implications on the clinical management of these patient groups. In fact, while the H_EGFr_ group would benefit from an increase of the dose/fraction (Hypo-fractionation) and the consequent reduction of OTT to compensate for the increase in the proliferation rate - corresponding to a reduced *T*_*D*_ -, the L_EGFr_ group does not require a reduction of OTT for which it would be more indicated a reduction of the dose/fraction (Hyper-fractionation) which would result in a reduced toxicity for all the organs at risk.

## Discussion

In the recent years there has been a great interest to find factors that predict tumours suitable for accelerated radiotherapy and considerable interest has been given to cell kinetic parameters such as the *T*_*pot*_. Since regeneration and tumour cell proliferation are mechanisms at the cellular level, particular attention has been focused on identifying the specific cellular characteristics, such as variations in EGFr expression. The latter is an important mediator of cell growth and its over-expression has been associated with tumour progression and poor survival in many solid cancers. Several studies have demonstrate the potential of EGFr as a predictive and prognostic marker in radiotherapy for HNSCC [19].

In the present study a direct demonstration of the link between EGFr status and the time factor in fractionated radiotherapy, has been made. All the clinical studies surveyed, from the available literature, had a random allocation for “reduced” or “conventional” OTT and demonstrated an increase in LCR when the OTT was reduced.

Unfortunately, OTT reduction yields clinical benefits in terms of LCR but could worsen the radiation-induced acute side effects which need to be carefully evaluated using appropriate radiobiological models [20].

Moreover, some studies also demonstrated that tumours with high EGFr respond better to the reduction of the OTT compared to low EGFr tumours [6,7,9]. The response was heterogeneous if referring to the sub-sites included in the analysis.

Therefore, our intent was to evaluate the extent of accelerated proliferation due to an EGFr over-expression, in terms of reduced actual doubling time as well as required dose to offset the effect of proliferation occurring in one day.

To obtain these results no assumption was made with the exception of the validity of a linear quadratic and *TCP* model. Therefore, the fact that the EGFr expression changes the radiosensitivity and the proliferation rate of the cells, has to be necessarily included in these models as a variation of the parameters (*α*, *β* and *T*_*D*_ ) describing them.

Although in all the studies selected for the survey, the accelerated repopulation of tumour cells during radiotherapy was suggested as an important cause of treatment failure, the main difficulty in our analysis was that head and neck cancer represents a heterogeneous group of cancers and the benefit is not act equal for the different tumours. We also attempted to estimate these differences.

We are aware that an important drawback in the analysis is to be found the differences among treatment modalities. Some studies have included radiotherapy alone, others postoperative radiotherapy, others the use of radio-sensitizing hypoxic drugs. We therefore stress the versatility and enormous potential of the method we propose.

Indeed, the relationship between elements representative of the radiation effects calculated only on groups of patients who undergo the same treatment, is based accordingly. In other words, although radiotherapy alone is profoundly different from postoperative radiotherapy or from radiotherapy combined with radio-sensitizing drugs, the relationship of the effects calculated within the same type of therapy, nullify these contributions, allowing to obtain only those due to the different expression of EGFr.

The validity of this statement is confirmed by the very low dispersion of data around the linear regression lines obtained from them. Our results clearly demonstrate proportionality between differences in treatment duration and correspondent ratios of LCR (the latter in logarithmic form). This strong linearity allowed us to quantify the reduction of actual doubling time of the H_EGFr_ group with respect to the L_EGFr_ group.

Unfortunately, in the papers, different definitions for the level of cut-points of EGFr expression were used. In some studies a cut-point of 50% was chosen as being objective and reproducible, others fixed a cut-point of 33%, 40%, 80%, etc. However, it is obvious that no dichotomous division between high and low EGFr tumour expression exist and a continuous variable must apply. In addition, because samples for the various studies were collected from various pathology departments and staining intensity can be dependent on tissue fixation [21], the evaluation of staining intensity was not entirely homogeneous. This was certainly the greatest source of approximation in the quantitative results obtained.

Despite these limitations, our results indicate a clear reduction of effective doubling time *T*_*D*_ in H_EGFr_ with respect to the L_EGFr_ groups. This reduction did not so excessive as the necessary to reach the minimum value which is represented by *T*_*pot*_ (that is the limit where the cell loss fraction is reduced to zero and the proliferation is fastest).

We consider this result very important, especially because an accurate estimate of *T*_*D*_ allows to obtain the equivalent dose for accelerated repopulation that is essential to making rational adjustments to the overall dose when the overall time is increased. This has become more than just an academic question in the area of IMRT when, instead of using shrinking field techniques, radiation oncologists commonly use a differential dose per fraction to deliver graded doses in the same overall treatment time.

Our results consisted of *D*_*prolif*_ systematically lower than those accepted in the literature that are often obtained through an evaluation of-the potential doubling time, which is a characteristic of each proliferative cell, and not through the effective doubling time, which is-a characteristic of a group of cells [22].

In the case of oropharynx, for example, we obtained values up to 0.39 Gy/day (0.31-0.47)_95%_ while in the literature we have values between 0.48-0.68 Gy/day [23,24]. For the larynx we obtained values up to 0.16 Gy/day (0.13-0.19)_95%_, while other estimates for this tumour are between 0.3 and 0.5 Gy/day [17]. Only in the case of hypopharynx we had values greater than 0.5 Gy/day (0.40-0.59)_95%_.

The difference can partly be explained by the heterogeneous behavior of the different sub-sites involved in our analysis (a specific sub-site clinical study could discriminate more finely between different contributions). However, our opinion is mainly based on the interpretation of the correlation between *T*_*D*_ and *T*_*pot*_.

As a first hypothesis, the reduction of *T*_*D*_ can be easily explained with a correspondent reduction of *T*_*pot*_, but clinical data has shown that for patients with short *T*_*pot*_ (fast tumours) there was no statistically significant trend to do worse [25]. Moreover, we found a reduction of *T*_*D*_ with an average factor of about 7 in the H_EGFr_ with respect to the L_EGFr_ group, and the same extent was never found by measures that assess *T*_*pot*_ from biopsy among patients.

Consequently, given that a shorter *T*_*D*_ may also result from a reduced *ϕ* after the beginning of treatment (see Appendix C), our results suggest that the latter possibility is favored. In this case, the tendency of *ϕ* toward zero, indicates a reduction of the clonogen doubling time *T*_*D*_ until it equals the pretreatment *T*_*pot*_. Hence, our results for *T*_*D*_ can be easily explained from an incomplete reduction of *ϕ* toward zero.

Furthermore, a *ϕ* reduction being associated with a low differentiation, would correspond to an increase in a non-differentiated component.

Thus, the question arises about how two different results may be reconciled.

On one hand, the simultaneous expression of a differentiated pattern and high levels of EGFr display a higher degree of accelerated repopulation compared to carcinomas with low levels of EGFr or poor differentiation [5]. On the other hand, as is clear from the Steel’s formula, the reduction of *T*_*D*_ is due to a reduction of the differentiation levels.

A possible explanation could be that two different levels of differentiation may coexist locally.

This hypothesis is based on the clinical observation that high levels of EGFr expression were found to be more pronounced at the tumour borders compared to the central parts of the tumour tissue (*p <* 0.0001) [6]. Therefore, on the border of the tumour, the EGFr over-expression would be compatible with a low level of differentiation and rapid tumour growth (as from Steel’s formula). In more central tumour areas, the low EGFr expression may be compatible with a high level of differentiation and reduced tumour cell proliferation. This spatial non-uniformity, suggests that the precise location of biopsy sampling and a subsequent classification of tumours (high or low EGFr and level of differentiation) are crucial. A such hypothesis, of course, requires further investigation in clinical studies.

## Conclusion

Increased expression of the EGFr can lead to enhanced proliferation which can be countervailed by reducing the time available for tumour cell proliferation, thereby reducing the overall treatment time. In this case, the impact of high EGFr expression changes from being a negative to a positive prognostic value in terms of local control rate.

In this study we introduced a model that allows to quantify the influence of EGFr expression in terms of reduced doubling time during the treatment and also the dose per fraction to be taken into account when the overall treatment time is reduced in accelerated radiotherapy. Furthermore, using this model, we can also estimate the parameters inherent in different sub-sites which may identify the optimal dose fractionation regime more likely to benefit these sub-sets of patients.

## Appendix A

To simplify the radiobiological analysis, eq. (1) can be rewritten by considering the *BED* as the difference between a dosimetric and a temporal component:

$$BED=BED\left(d\right)-BED\left(T\right)$$

The *BED(d)* for high EGFr expression group, for instance, is [26].

$$BED_{H}\left(d\right)=nd\left(1+\frac{d}{\alpha_{H}/\beta_{H}}\right)$$

while the *BED(T)* for the same group is

$$BED_{H}\left(T\right)=\frac{\ln2}{\alpha_{H}}\frac{\left(T-T_{K}\right)}{T_{d}^{H}}=\frac{\gamma_{H}}{\alpha_{H}}\left(T-T_{K}\right)$$

Thus, to take into account the differences of radiosensitivity as well as OTT, two and four possible expressions of *BED(d)* and *BED(T)* are considered, respectively:

$$\begin{array}{l} {\text{BED}}_{H}\left(d\right),{\text{BED}}_{L}\left(d\right),{\text{BED}}_{H}\left(T^{S}\right),{\text{BED}}_{H}\left(T^{F}\right),\\ \;{\text{BED}}_{L}\left(T^{S}\right),{\text{BED}}_{L}\left(T^{F}\right) \end{array}$$

For different EGFr expressions and OTT we have also

$$LCR_{H}^{F},LCR_{H}^{F},LCR_{L}^{S},LCR_{L}^{F}$$

where, for example

$$LCR_{H}^{S}=\exp\left\lfloor -\rho V\exp\left(\alpha_{H}\left(BED_{H}\left(d\right)-BED_{H}\left(T^{S}\right)\right)\right)\right\rfloor$$

Therefore, by taking the natural logarithms of this expression and dividing it for the same expression with a different OTT, we can nullify the contributions of *ρV* and *BED(d)* – because of the same fractionation – obtaining only those due to the different expression of EGFr with respect to the OTT.For the high EGFr expression group, for instance, we have

$$\begin{array}{l} \ln LCR_{H}^{S}/\ln LCR_{H}^{F}=\exp|\alpha_{H}(BED_{H}\left(T^{S}\right)\\ \;-BED_{H}\left(T^{F}\right))| \end{array}$$

from which, by taking the natural logarithms again, we have eq. (3.a). The same procedure leads to eq. (3.b).

## Appendix B

The equations (3.a) and (3.b) are valid if the hypothesis of equal dosimetric component of *BED* in conventional and accelerated fractionation, is valid. However, this is not true for all the papers in the literature surveyed. The differences in terms of dose fractionation were corrected using the follows

$$\ln LCR_{c}=e^{-a\left(BED^{S}\left(d\right)-BED^{F}\left(d\right)\right)}\ln LCR_{\mathrm{nc}}$$

where the indexes *nc* and *c* stand for “*non-corrected*” and “*corrected*”, respectively. *BED*^*S*^*(d)* and *BED*^*F*^*(d)* refer to different *BED* dosimetric components in conventional and accelerated fractionation, respectively. The exponential factor incorporates the difference of *BED* only due to the dosimetric *BED* component, and therefore enables the contribution to be corrected, thanks to this component.

## Appendix C

In order to estimate the actual doubling time for each sub-site in the analysis, different potential doubling times *T*_*pot*_ were considered from literature [15]. The latter has been introduced by Steel as the clonogen doubling time that would be measured if cell loss was ignored, i.e. if both daughter cells remained clonogenic after mitosis [14].In practice clonogens are lost through many possible mechanisms, including differentiation, death, and metastasis, and the net result is that *T*_*D*_ will be longer than *T*_*pot*_.Steel’s formula can be written as follows:

$$T_{D}=\frac{T_{\mathrm{pot}}}{1-\phi }$$

wherein *ϕ* is the cell loss factor. This equation shows that *T*_*D*_ can be calculated if *T*_*pot*_ and *ϕ* are known.In particular *T*_*pot*_ can be measured by a single biopsy using flow cytometry or immunohistochemistry techniques, while the average cell loss factor < *ϕ* > was obtained in our analysis taking the average potential doubling time weighted on percentages for any sub-site (*<*^*i*^*T*_*pot*_ *> =∑*_*i*_*p*_*i*_*·*^*i*^*T*_*pot*_ with *i = 1,.,4* and *p*_*1*_ = 21% for oral cavity, *p*_*2*_ = 20% for oro-pharynx, *p*_*3*_ = 17% for hypo-pharynx and *p*_*4*_ = 42% for larynx), by the follows

$$<\phi >=1-\frac{{<}^{i}T_{\mathrm{pot}}>}{{<}^{i}T_{D}>}$$

The actual doubling time for each *i* sub-site, was then obtained as follows

$${}^{i}T_{D}=\frac{{}^{i}T_{\mathrm{pot}}}{1-<\phi >}$$

Results were reported in Table 3.

## Abbreviations

(BED): Biologically Effective Dose; (D_prolif_): Dose required to offset the proliferation occurring in one day; (EGFr): Epidermal Growth Factor Receptor; (FCM): Flow Cytometry; (ϕ): Cell Loss Factor; (H_EGFr_): High EGFr expression group; (Hi): Immunohistochemistry; (HNSCC): Head and Neck Squamous Cell Carcinoma; (LCR): Local Control Rate; (L_EGFr_): Low EGFr expression group; (OTT): Overall Treatment Time; (TCP): Tumour Control Probability; (T_D_): Effective Doubling Time; (T_k_): Time of onset of accelerated proliferation; (T_pot_): Potential Doubling Time.

## Competing interests

The authors declare they have no competing interests.

## Authors’ contributions

PP developed the model, designed the study and wrote the manuscript. AN, LS, BAJ, DA, MC, FB, RC, AF and GI checked the goodness of the study from oncology, radiotherapy and mathematical points a view. RC made the graphical illustrations. GS, MB, RO and MS supervised the manuscript from radiobiological and clinical points a view. All co-authors approved the manuscript.
